# Draft Genome Sequence of Rhizobium sophoriradicis H4, a Nitrogen-Fixing Bacterium Associated with the Leguminous Plant Phaseolus vulgaris on the Coast of Peru

**DOI:** 10.1128/genomeA.00241-18

**Published:** 2018-05-24

**Authors:** Ernesto Ormeño-Orrillo, Yohana Aguilar-Cuba, Doris Zúñiga-Dávila

**Affiliations:** a Laboratorio de Ecología Microbiana y Biotecnología, Departamento de Biología, Facultad de Ciencias, Universidad Nacional Agraria La Molina, Lima, Peru

## Abstract

The genome sequence of Rhizobium sophoriradicis H4, a nitrogen-fixing bacterium isolated from the common bean (Phaseolus vulgaris) in Peru, is reported here. The genome assembly revealed a 6.44-Mbp genome which was distributed into 95 contigs, with *N*_50_ and *L*_50_ values of 293 kbp and 9, respectively. The genome contained 6,312 coding sequence (CDS) genes and 52 RNA genes (49 tRNAs and 3 rRNAs).

## GENOME ANNOUNCEMENT

Rhizobium sophoriradicis is an alphaproteobacterial species first isolated in China as a root-nodule symbiont of the leguminous plant Sophora flavescens (1). This rhizobium was later found to be associated with the common bean (Phaseolus vulgaris) in Iran (2) and South Africa (3). During a study of the rhizobial diversity of common bean symbionts on the coast of Peru, we found that R. sophoriradicis is present in some areas where this leguminous plant is cultivated. Here, we report the genome sequence of a Peruvian strain of this species.

Sequencing was performed using the Illumina MiSeq platform with 300-bp paired-end reads. Reads were quality trimmed with Trimmomatic (4) and assembled with SPAdes (5). Genome completeness was evaluated using the program BUSCO (6). The sequences were sent to the Rapid Annotations using Subsystems Technology (RAST) server (7) for functional annotation. The genome assembly of R. sophoriradicis H4 consisted of 95 contigs ranging in size from 237 bp to 539,995 bp, with a mean coverage of 122×. The *N*_50_ and *L*_50_ values were 293 kbp and 9, respectively. A completeness score of 100% was obtained for the assembly, indicating that all of the genome of strain H4 was recovered. The genome size was estimated at 6.44 Mbp, and the GC content was 61.4%. The number of predicted CDS genes was 6,312, while the RNA genes included 49 tRNAs and 3 rRNAs.

Functions could be assigned to 74% of the CDS genes of R. sophoriradicis H4. An abundance of genes involved in the metabolism of carbon and nitrogen sources revealed that strain H4 is a metabolically versatile bacterium. The traits related to plant root colonization encoded in its genome included flagellar motility, chemotaxis, surface adhesion via a type IV pilus, siderophore production and uptake, type VI secretion, and exopolysaccharide biosynthesis.

When aligned against symbiotic plasmids of other rhizobia, R. sophoriradicis H4 contigs showed high homology to symbiovar phaseoli plasmids. Within these contigs, we found all the nodulation and nitrogen fixation genes required to establish a successful symbiotic relationship with legumes. Among nodulation genes, we found *nodZ*, *noeI*, and *nolL*, whose presence indicates that nodulation factors produced by strain H4 bear methylated and acetylated fucose residues at the reducing end, while genes *nolO*, *nodS*, and *nodU* indicate methyl and carbamoyl decorations at the nonreducing end (8). Also, putative symbiotic plasmid contigs included genes for a type III secretion system, which may be required for an optimal association (9), a type IV secretion system probably for conjugal transfer of the symbiotic plasmid (10), an uptake ABC transporter for nopaline, which may confer competitive ability (11), the genes *teuBAC_1_C_2_*, required for utilization of root exudates (12), and genes for the biosynthesis of gibberellins.

This study reports the first genome sequence of a rhizobial symbiont of the common bean isolated in Peru, which is also the first genome sequence of a strain of the R. sophoriradicis species.

### Accession number(s).

The nucleotide sequence for strain H4 has been deposited in GenBank under the accession number PSOW00000000.
